# Laparoscopic pectopexy: initial experience of single center with a new technique for apical prolapse surgery

**DOI:** 10.1590/S1677-5538.IBJU.2017.0070

**Published:** 2017

**Authors:** Ahmet Kale, Alper Biler, Hasan Terzi, Taner Usta, Ebru Kale

**Affiliations:** 1 Department of Obstetrics and Gynecology, Kocaeli Derince Training and Research Hospital, Kocaeli, Turkey; 2 Department of Obstetrics and Gynecology, Tepecik Training and Research Hospital, Izmir, Turkey; 3 Department of Obstetrics and Gynecology, Bağcılar Training and Research Hospital, Istanbul, Turkey; 4 Department of Biochemistry, Kocaeli Derince Training and Research Hospital, Kocaeli, Turkey

**Keywords:** Laparoscopy, Pelvic Organ Prolapse, Vagina

## Abstract

**Objective:**

To share our first experience with laparoscopic pectopexy, a new technique for apical prolapse surgery, and to evaluate the feasibility of this technique.

**Materials and Methods:**

Seven patients with apical prolapse underwent surgery with laparoscopic pectopexy. The lateral parts of the iliopectineal ligament were used for a bilateral mesh fixation of the descended structures. The medical records of the patients were reviewed, and the short-term clinical outcomes were analyzed.

**Results:**

The laparoscopic pectopexy procedures were successfully performed, without intraoperative and postoperative complications. De novo apical prolapse, de novo urgency, de novo constipation, stress urinary incontinence, anterior and lateral defect cystoceles, and rectoceles did not occur in any of the patients during a 6-month follow-up period.

**Conclusion:**

Although laparoscopic sacrocolpopexy has shown excellent anatomical and functional long-term results, laparoscopic pectopexy offers a feasible, safe, and comfortable alternative for apical prolapse surgery. Pectopexy may increase a surgeon's technical perspective for apical prolapse surgery.

## INTRODUCTION

Pelvic organ prolapse (POP) affects millions of women worldwide, and is a health problem for 50% of parous women aged over 50 years (1). At the same time, the number of surgical procedures performed for prolapse has increased enormously in recent years, as a result of changes in population distribution; while 12.7% of women in the United States were aged over 65 in 2000, this figure will rise to 20% by 2030 (2). Similarly, the percentage of women in Germany aged over 65 was 20% in 2011, and this proportion will increase to 35% by 2060 (3).

Apical prolapse refers to the downward displacement of the vaginal apex, uterus, or cervix. It may be associated with various signs and symptoms, including vaginal bulging, palpable or visible tissue protrusion, pelvic pain, dyspareunia, or obstructed intercourse. Women with apical prolapse often experience altered bladder and bowel functions, such as irritative or obstructed voiding, urinary retention or urinary incontinence, obstructed defecation, and fecal urgency or fecal incontinence (4).

Numerous previous studies have shown that sacrocolpopexy or sacrouteropexy represents the most effective option for apical prolapse surgery (4–6); sacrocolpopexy remains the most suitable surgical procedure for restructuring the physiological axis of the vagina (4–6). In contrast with abdominal sacrocolpopexy, laparoscopic and robot-assisted approaches avoid the need for a large abdominal incision and minimize bowel manipulation, potentially leading to less postoperative pain and a shorter recovery time (7, 8).

Although sacrocolpopexy has been the most effective option over time, the procedure is still associated with some problems, and the most frequently reported complications include defecation disorders and stress urinary incontinence (SUI) (9). Previous studies have consistently reported that gastrointestinal complications, such as small bowel obstruction, ileus, or defecation disorders occur in approximately 0.1 to 5% of sacrocolpopexy procedures. The mesh placed between the sacrum and vagina (cervix) always narrows the pelvis, and the cause of the defecation disorders may be reduced space in the pelvis (outlet obstruction), adhesions, or trauma of the hypogastric nerves (5, 7, 9–11). However, presacral hemorrhage is the most worrying intraoperative complication of sacrocolpopexy, and may have life-threatening consequences (11).

POP is more associated with obese patients (12), and the advantages of laparoscopic surgery are more important for this patient Group. However, this method may be restricted, due to the difficulty of the surgical field. In 2007, Banerjee and Noe described a new method of endoscopic prolapse surgery that was especially developed for obese patients, in which the lateral parts of the iliopectineal ligament are used for bilateral mesh fixation of the descended structures (13). In this method, the mesh follows round and broad ligaments without crossing the ureter or bowel; therefore, the pelvic outlet does not shrink. In addition, the hypogastric vessels are also a safe distance from any danger (13).

**Figure 1:**
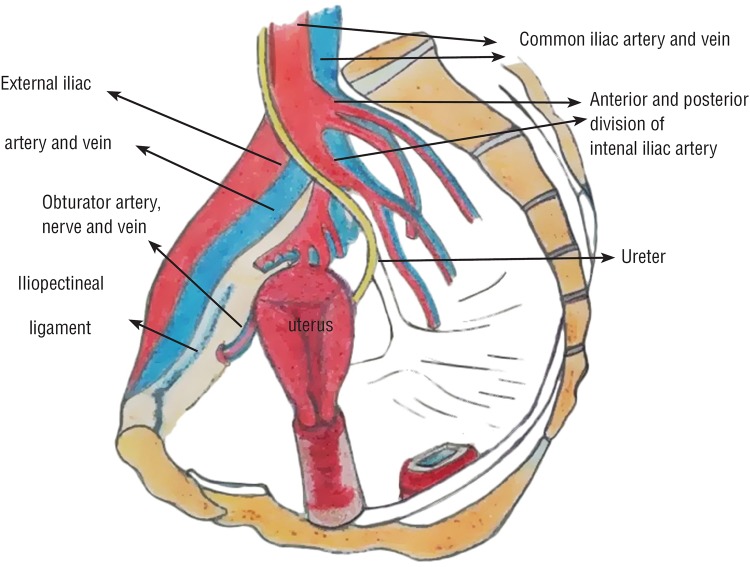
Anatomic details of the iliopectineal ligament.

The ilipectineal ligament is an extension of the lacunar ligament that runs on the pectineal line of the pubic bone (14) (Figure-1), and is significantly stronger than the sacrospinous ligament and the arcus tendineus of the pelvic fascia (15). The structure is strong, and holds suture well. It is also possible to find sufficient material for a suture in the lateral part of the iliopectineal ligament, facilitating reconstruction of the pelvic floor (16). This segment of the ligament is situated at the second sacral vertebra (S2) level which is the optimal level for the physiological axis of the vagina. S2 level is the anchor point for the physiological axis of the vagina (16).

We recently successfully performed prolapse surgery in seven patients, without any complications, using a new laparoscopic pectopexy technique. This was the first short-term Turkish experience with this technique, and it is described and shared in this paper.

## MATERIALS AND METHODS

A total of seven women who underwent laparoscopic pectopexy between May 2014 and January 2015 at the Kocaeli Derince Educational and Research Hospital, Kocaeli, Turkey, were included. The patients presented with either symptoms related to apical prolapse, such as sensation of pressure on the vagina, seeing or feeling a bulge/protrusion, lower back pain, dyspareunia, and other sexually related symptoms, or associated urinary symptoms, such as incontinence, frequency, urgency, and urinary retention. All operations were performed by the same surgical team, and all patients underwent surgery after their informed consent was obtained. The study was approved by the Ethics Committee of the Kocaeli Derince Educational and Research Hospital (Registration number KÜ GOKAEK 2016/204).

The extent of the genital prolapse was assessed not only by a gynecological examination, but also via ultrasonography. The pelvic organ prolapse quantification system (POP-Q) for prolapse assessment was used. In order to assess the influence of pressure, the patients were examined both in a lying and in a sitting position; this assessment was important to avoid an over-or under-correction. Only symptomatic primary vaginal or uterine prolapse patients with POP Q II and above were included. Exclusion criteria were previous operations for vaginal prolapse correction, pelvic inflammatory disease, and previously identified, or strongly suspected, massive adhesions in the pelvic cavity.

The patient's medical records and video recordings of the operations were reviewed. All patients were analyzed in terms of age, body mass index (weight in kilograms divided by the square of the height in meters), estimated blood loss (EBL), operation time, intraoperative complications and postoperative complications.

The patients were followed up for at least 6 months after surgery, and the relapse occurrence of apical prolapse, anterior and lateral defect cystoceles, as well as the incidence of de novo urinary symptoms, rectoceles, and defecation disorders, were recorded. We used the defecation section of the International Consultation on Incontinence Ouestionnaire to document the defecation disorders.

### Surgical procedures

All the operations were performed under general anesthesia with endotracheal intubation, and standard intramuscular cephalosporin antibiotics were used for prophylaxis. The patients were then placed in a modified lithotomy position, with the hips at an approximate 180° extension, the knees flexed at almost 90°, and with the table tilted in a nearly 45° Trendelenburg position. Both arms were tucked along the patient's side. A 10mm trocar (Endo Ethicon) was inserted directly from the umbilicus, and pneumoperitoneum was generated until an intra-abdominal pressure of 14mmHg was achieved. Three additional 5mm ports were inserted under direct visualization of the lower intra abdominal area; median, left, and right from 2 cm medial and superior to the anterior superior iliac crests. Following sterilization of the skin and covering of the patient, a RUMI^©^ uterine manipulator with a Koh Cup™ colpotomizer (Cooper Surgical; Trumbull, Connecticut, US) was trans-vaginally introduced at the beginning of the procedure. The surgeon stood on the patient's left, and the first assistant handled the scope on the patient's right. The second assistant was positioned between the legs of the patient. Operation time began with the first skin incision and ended with the final closure of an incision.

### Pectopexy technique

**Figure 2:**
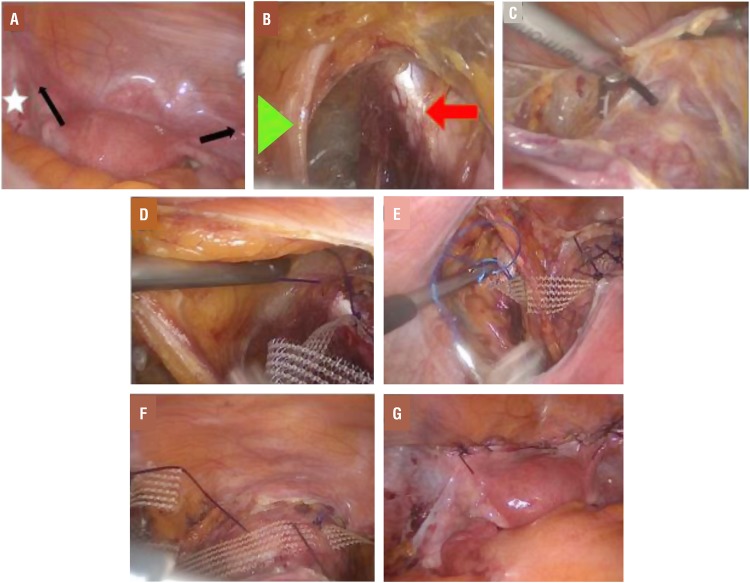
Intraoperative stages. Determination of the round ligaments (arrows) and external iliac vessels (star). The peritoneal layer is opened along the right round ligament toward the pelvic side wall. Soft tissue in this area was dissected with blunt dissection. The iliopectineal ligament (arrow) and the medial umbilical ligament (triangle) are demonstrated. The same procedure is then repeated on the left side of the patient. The peritoneal layers on both sides are opened toward the cervix. After completion of dissections, the ends of the mesh are sutured to both iliopectineal ligaments via the intracorporeal suture technique, using nonabsorbable sutures. The middle of the mesh is fixed at the lower anterior segment of the uterus with three stitches. The peritoneum above the mesh is sutured with an absorbable suture material.

We performed this procedure as previously described by Banerjee and Noe (13). First, we opened the peritoneal layer along the right round ligament toward the pelvic side wall (Figure-2A). An incision in the medial and caudal direction was made with an Harmonic scalpel, and the right external iliac vein was visualized. Soft tissue in this area was dissected with blunt dissection, so an approximately 4-5 cm segment of the right iliopectineal ligament (Cooper ligament) adjacent to the insertion of the iliopsoas muscle could be identified (Figure-2B). The same procedure was then repeated on the left side of the patient. The peritoneal layers on both sides were opened toward the vaginal apex, and the anterior and posterior areas of the vaginal apex were prepared for the mesh fixation. In patients with a preserved uterus, the anterior peritoneum of the uterus was dissected, and the lower anterior segment of the uterus was prepared for the mesh fixation (Figure-2C). After completion of dissections, a polyvinylidene fluoride monofilament mesh (DynaMesh^©^ PVDF, 3×15 cm) was inserted into the abdominal cavity. The ends of the mesh were sutured to both iliopectineal ligaments via the intracorporeal suture technique, using nonabsorbable sutures (Figures 2D and 2E). The mesh in the tension-free position was fixed to the vaginal apex or uterus with polydioxanone sutures (Figure-2F), and the vaginal apex or uterus was provided with a hammock-like fixation. Finally, the peritoneum above the mesh was sutured with an absorbable suture material (Figure-2G).

Low-dose vaginal estriol treatment was postoperatively initiated, and it was recommended that all patients continue with this for at least 6-8 weeks following the procedure. We also advised the performance of regular pelvic floor exercises to provide adequate healing and scar tissue formation.

### Statistical analysis

Statistical analysis was carried out using Statistical Package for the Social Sciences software, version 20.0 (SPSS Inc., Chicago, Illinois, USA), and descriptive statistics were used to describe the study. Data were expressed as number and percentage or mean with standard deviation.

## RESULTS

Over the study period, seven patients underwent laparoscopic pectopexy procedures. EBL was no more than 50 mL and operation time was no longer than 80 minutes (Table-1). Laparoscopic pectopexy was successfully performed, without intraoperative or postoperative complications. Conversion to laparotomy was not required in any of the cases and nor were postoperative blood transfusions. The patients remained in hospital for a maximum of 24 hours and were discharged in good health. The therapy satisfaction rates were high in all patients, who were followed up in the outpatient clinic at 1 week and then at 6 months after discharge. De novo apical prolapse, de novo urgency, de novo constipation, SUI, anterior and lateral defect cystoceles, and rectoceles did not occur in any of the patients during the 6-month follow-up period.

**Table 1:** Details of laparoscopic pectopexy procedures.

Patient no	Age (years)	BMI (kg/m^2^)	Pelvic examination	Operation time (min.)	EBL (mL)	Intraoperative Complications	Postoperative Complications
1	67	23.4	Vaginal vault prolapse	80	40	None	None
2	56	20.5	Vaginal vault prolapse	74	20	None	None
3	59	23.6	Vaginal vault prolapse	60	30	None	None
4	61	22.9	Vaginal vault prolapse	70	35	None	None
5	50	19.5	Vaginal vault prolapse	72	40	None	None
6	40	23.5	Uterine prolapse	55	50	None	None
7	39	24.4	Uterine prolapse	59	45	None	None

**BMI** = body mass index; **EBL** = estimated blood loss.

## DISCUSSION

Several previous studies have shown that both abdominal and laparoscopic sacrocolpopexy for apical prolapse surgery is related to excellent anatomical and functional outcomes in long-term follow-up (4–6, 17). Apical prolapse repair has been performed laparoscopically for over 20 years (10, 18), and, although it depends on the surgeon's ability, potential problems may arise during laparoscopic sacrolcolpopexy; the sigmoid is retracted to the left, allowing identification of the sacral promontory, and when working in the area of the sacrum, care should be taken to avoid damage to the sigmoid, presacral veins, and right ureter. Another issue is accessibility to the surgical area at the ventral side of the sacrum; therefore, many surgeons have modified the technique and have fixed the mesh to the top of the promontory. However, this change of mesh localization results in a positional change in direction to the abdominal wall (10, 16).

Laparoscopic pectopexy is a new type of endoscopic prolapse surgery. It uses the lateral parts of the iliopectineal ligament for a bilateral mesh fixation of the descended structures, so fewer potential long-term problems are expected (19). The pelvic outlet does not narrow with this procedure, as is expected with sacrocolpopexy, and, compared to the latter, laparoscopic pectopexy is not associated with a high intraoperative risk (19). In the present study, there were no intraoperative complications or postoperative complications. Noe et al. compared the laparoscopic pectopexy and sacrocolpopexy procedures in a randomised comparative clinical trial that was conducted in 83 patients who had only symptomatic primary vaginal prolapse POPQ ≥2 (19). They showed that mean operation time and blood loss were reduced in the pectopexy Group.

The incidence of de novo SUI following sacrocolpopexy is 15.9-37.6% (17, 20, 21). North et al. reported de novo SUI in half of women without concomitant continence surgery with sacrocolpopexy (18). In contrast with other studies, Noe et al. observed de novo SUI in approximately 5% of women in both laparoscopic pectopexy and laparoscopic sacrocolpopexy groups (16). Although we are not capable of analyzing the long-term outcomes of the patients in the current study, no occurrences of de novo SUI were recorded.

One important problem that is observed following sacrocolpopexy is that of gastrointestinal complications; defecation problems, particularly constipation, are most common (18, 22, 23). As expected, Noe et al. showed a statistically significant difference in the incidence of de novo defecation problems following laparoscopic pectopexy and sacrocolpopexy- 0% and 19.5%, respectively (16). In accordance with these results, we did not observe defecation problems. This may be explained by the fact that pectopexy neither reduces the space of the pelvis (outlet obstruction) nor carries the risks of trauma to the hypogastric nerves.

It has been reported that this technique may be protective against de novo anterior and lateral defect cystoceles, due to the lateral placement of mesh (16). In our study, de novo lateral defects were not observed.

We successfully performed laparoscopic pectopexy procedures in seven patients, without intraoperative and postoperative complications. As already mentioned, de novo apical prolapse, de novo urgency, de novo constipation, SUI, anterior and lateral defect cystoceles, and rectoceles did not occur in our patients during the 6-month follow-up period. However, the number of cases included was one of the main limitation of this study. Besides there was no control group to compare the results.

We believe that laparoscopic pectopexy offers several practical advantages: (1) it enables the surgeon to use a wide area in the pelvis, that reacts more satisfactorily in complex surgical conditions; (2) it does not reduce the pelvic space, so postoperative defecation and urinary disorders are not expected; (3) the iliopectineal ligament is very strong, thus it is expected that there will be a very low rate of postoperative recurrence of apical prolapse; (4) the iliopectineal ligament fixation of apical prolapse does not change the physiologic axis of the vagina because S2 level is the anchor point for the physiological axis of the vagina; and (5) the iliopectineal ligament is far from the ureter, intestines, sigmoid, and presacral veins. During surgery, there is very little damage to these structures, so the iliopectineal ligament is a safe area for apical prolapse reconstructive surgery.

## CONCLUSIONS

We have shown in our study that pectopexy may be a feasible, safe, and comfortable procedure that can be performed in the apical prolapsus surgery. Laparoscopic pectopexy might be an alternative technique to sacrocolpopexy. However, this case series displays the initial experience of a new procedure, and further prospective comparative studies are necessary to show long-term effectiveness.
